# Changing Employment and Work Schedule Patterns over the 30 Working Years—A Sequential Cluster Analysis

**DOI:** 10.3390/ijerph192013677

**Published:** 2022-10-21

**Authors:** Wen-Jui Han, Julia Shu-Huah Wang

**Affiliations:** 1 Silver School of Social Work, New York University, New York, NY 10003, USA; 2 Department of Social Work, National Taiwan University, Taipei 10617, Taiwan

**Keywords:** employment patterns, longitudinal, NLSY79, sequence analysis, work schedules

## Abstract

Objective. As labor markets have become increasingly volatile and precarious since 1980s, more workers are susceptible to working conditions such as unpredictable and unstable hours, threatening their economic security. However, our understanding of employment patterns regarding the changes in work schedules over our working lives has yet been established. This study builds our knowledge in this area by paying attention to how social positions may shape the specific work schedule patterns over our working lives. Methods. We used the National Longitudinal Survey of Youth-1979 (NLSY79) to examine our research questions. NLSY79 follows a nationally representative sample of United States men and women between the ages of 14 and 22 when first interviewed in 1979. The participants were then interviewed annually until 1994 and then biennially thereafter. We first conducted a sequence analysis to examine work schedule patterns between ages 22 and 53 (*n* = 7987). We then used a multinomial logit regression to examine the factors contributing to specific work schedule patterns, with attention to social position. Results. Our sequence analysis results suggest five work schedule patterns during 31 years of adult life: working only standard hours (25%), mainly standard hours with some portions of nonstandard hours (38%), standard hours during early working years but transitioning to either largely variable or mainly evening or night hours (14% and 13%), and mostly not working (10%). Our multinomial logit analysis indicates that being non-Hispanic Black, having a high school degree or below, or having ever experienced poverty or welfare by age 23 were more likely to have a nonstandard work schedule pattern than their counterparts. Conclusions. Our analysis underscores the dynamic employment patterns over our working lives, with some groups more likely than others to be engaged in nonstandard or volatile work schedules. Importantly, the groups likely to be subject to nonstandard work schedules also tend to have relatively disadvantaged social positions, thus worsening their vulnerability in securing work characterized by stability and economic security.

## 1. Introduction

Throughout a person’s lifetime, combating idleness and fulfilling wants and needs are critical not only to human survival but also to sustaining a decent living standard [1]. Employment that offers a living wage and working conditions conducive to a fulfilling life is central to survival and well-being. People pursue and obtain work based on their interests, their skills and experience, job availability, and personal preferences (e.g., competing demands between caregiving, school, and leisure). However, around the globe, the technological and digital revolution since the 1980s has transformed how we live and work, with implications for not just our economic well-being, but also for our health and social well-being [2]. The standard form of employment featuring full-time, year-round work with decent job-associated benefits has long ago been replaced by one characterized by precarity [3,4]. Many workers today are forced to take low-wage, low-skilled jobs with unpredictable and unstable hours often falling outside daytime hours [3,5]. Indeed, work schedules (e.g., early mornings, evenings, nights, rotation shifts) have become a staple feature of a precarious job for these jobs also tend to be low-wage and low-skill [5]. In addition to examining the employment trajectories across one’s working years that is primarily based on predominately samples of White men, the extant research has also examined the nuances of work pathways and patterns by gender [6] and by race-ethnicity [7,8]. However, we do not yet have a solid understanding of the overall work schedule patterns throughout the working years, a critical aspect of a precarious job.

A large body of scholarship has shown the importance of work schedules to the well-being of individuals [2,5,9] and their family members [10]. The seminal work of Harriet Presser [5] established who is most likely to be engaged in work that requires hours outside of the regular daytime hours: primarily young people, racial and ethnic minorities, those with low educational attainment, and single parents. These sociodemographic groups are also likely to hold precarious jobs; jobs tend to low-wage, low-skill, and high insecurity (few or no job benefits with a high likelihood of unstable employment) [4]. This study aims to extend Presser’s work documenting the snapshot of demographic compositions of people with nonstandard work schedules by using a person-driven statistical approach to provide a life-course look at work schedule patterns for a national representative sample (the National Longitudinal Study of Youth-1979) of about 7000 people in the U.S. over 30 years, from age 22 to age 53. This study focuses on workers in the United States concerning work schedule patterns throughout their working lives to inform our understanding of how our work has become ever more critical to our daily experience. Our work thus carries significant implications for our well-being on a global scale when the global market has become increasingly polarized [4].

### 1.1. The Labor Market Transformation in the United States

A recent report published by the U.S. Census Bureau documented a variety of employment patterns over the past 75 years, from 1939 to 2015 [11]. Specifically, from World War II until the 1950s, the number of manufacturing jobs grew substantially, accounting for the primary job gains in the United States. Thereafter, however, manufacturing sectors have suffered continuous job losses. Starting from the 1950s, private service-providing industries such as retail trade, professional and business services, and private education and health care saw the most job gains; these gains were particularly pronounced during the 1970s. Such jobs have continued to increase, though at a slower pace than between 1945 and the 1970s. Since the 1980s, the information sector, assisted by advanced technology, has seen steady growth. From the 1990s to the early 2000s, jobs related to information and technology expanded, amplified by globalization. The Great Recession of the late 2000s affected all industries, with varying degrees of slow job growth (in the service industry) and job loss (in the manufacturing sector).

These 75-year employment trends illustrate how the U.S. labor market has transformed from predominately manufacturing jobs with good benefits to more of a service economy, where low-wage, low-skill, and unpredictable and unstable jobs are the norm and on the rise. Technology and communications advancements have facilitated such a transformation and polarized the labor market regarding wages and jobs [12]. Specifically, computations and programming operations by machines have enabled rapid improvements in the productivity of information and communications technologies. As a result, the secular price decline in the actual cost of symbolic processing (e.g., microchips reduce the speed of literature search from days in a physical library to seconds online) creates vast economic incentives for employers to substitute information technology for expensive labor in performing workplace tasks [13]. Computational programming also allows many information-based tasks offshoring to foreign worksites with cheaper labor (e.g., remote customer service) [12]. Consequently, machines or communication technologies replaced many middle-skilled jobs (e.g., assembly-line workers and clerical) that used to bring decent wages with decent benefits before 1970s, creating a hollowing-out effect. The automation and offshoring of machine-operatable tasks, in turn, raises relative demand for workers who can perform tasks concerning abstract (e.g., high-skilled, problem-solving) and manual (e.g., food preparation, cleaning) that are complementary to functions performed by technology. The implications for the labor market and the observed changes are that technological advances favor high-skilled workers who tend to have high education (e.g., professionals who use computer-produced information to do problem-solving tasks), resulting in raising their wages. At the same time, by favoring high-skilled workers, technological advances reduce the wages of low-skilled workers (e.g., performing manual labor in the service sector) despite a high demand to serve the 24/7 economy. In addition, the advances in computational models to predict the flow of customers in retail stores have allowed employers to increasingly adopt just-in-time scheduling, producing unpredictable and unstable work hours for employees who need to be available with seconds of notice, despite such a scheduling practice hurts morale and does not produce savings [14]. Scholars have documented how this transformation has altered the lives of our workers to a worse instead of a better turn due to the rising demands of labors in low-wage and low-skill jobs with unpredictable, unstable, and nonstandard hours [3,5]. Globalization, while inevitable, may have exacerbated such trends [3,4].

At the same time, liberal labor policies since the late 1940s in the United States have provided employers with more tools to curtail workers’ union efforts. For example, the 1947 Taft-Hartley Act forced National Labor Relations Act (NLRA) to prioritize litigation against unions, thwarting workers’ efforts and rights to unionization [15]. Moreover, the NLRA’s efforts to hold employers accountable for violating workers’ unionization rights were stymied in the courts, in addition to a grossly insufficient penalty on employers when found violating the law [15]. The total effect of these trends produced by liberal labor policies and weakening union rights is manifested by the dramatic drop in unionization since the 1970s—from 35% in the 1970s to an all-time low of 10.5% in 2015, the end of the study period [3,16]. Historically, workers in the manufacturing sector tend to be protected by collective agreements with decent benefits, including health insurance, through their unions. The decline in the strength of unions, coupled with the growth of the service economy, which traditionally has weak or no unionization, has undermined worker protections, causing economic inequality to soar [3]. These forces were in evidence in the wake of the Great Recession. It took 51 months to recover the jobs lost during this most recent recession, the longest recovery period since 1939 [11]. Employment growth since 2007 has been concentrated in low-wage jobs (e.g., personal care, food preparation) and high-wage employment (e.g., computer science, management), causing a polarization of labor markets, attributed largely to information and technological shifts and globalization [3,17]. An extensive scholarship has demonstrated that the erosion of worker bargaining power, individually and collectively, has led to wage suppression and the deterioration of labor’s share of income [18]. Combined, these trends and changes created by technological advances and liberal labor policies have produced and accelerated the phenomenon of precarious employment, employment that is low-wage, low-skill, unpredictable, and nonstandard hours, with little or no benefits (e.g., health insurance).

### 1.2. Changing Employment Patterns over the Working Years

The documented 75-year labor market trends and transformations in the U.S. economy carry significant implications for people in different industries and occupations, with downward changes in employment prospects and economic security for some groups. A large body of scholarship has examined and refined our understanding of how both micro- and macro-level factors (e.g., gender, race-ethnicity, education, hiring practices, redlining policies) might shape social stratification and inequality; women, nonwhite workers, and those with little education have been particularly hard hit by these economic shifts [8,19,20]. Most studies have examined these issues either at one time point or over an extended period of one’s working years. When longitudinal data have been used, the focus has been chiefly on, for example, how wages and work hours over the working years might have shaped one’s economic security after retirement [21,22]. The field has also long acknowledged that early scholarship primarily used samples of predominately White, middle-class men, with little attention paid to other groups whose working life may be far from a stable work history with a clear beginning (upon receiving a high-school or college degree) and end (upon reaching retirement age) [21,22].

#### 1.2.1. Variation by Demographic Characteristics

Since the 1990s, a large body of scholarship has worked to address the early omissions, examining work trajectories and patterns by gender, race-ethnicity, and education, among other factors [6,20,23,24]. For example, nonwhite workers and female workers tend to follow a different employment path from White men, largely due to various structural constraints including higher risks of being unemployed and for an extended period, or of having access only to low-paying jobs with irregular hours and no benefits, such as health insurance.

In 1970, women’s labor force participation rates, particularly among White women, depended heavily on marriage and motherhood; their participation peaked at ages 20–24, fell at ages 25–34, and then gradually climbed to another peak at ages 45–54 before tapering off [25]. Since the 2000s, partly due to women’s rising educational achievement, this pattern of peaks and valleys is no longer evident; women’s labor force participation pattern has begun to resemble that of men, although at an overall lower participation rate [6,25]. However, women continue to face competing demands from family and work. Women thus have followed a somewhat different employment trajectory from men, being more likely to be concentrated in the service sectors with low wages, non-day time schedules, and part-time hours [6]. Part-time hours may allow women to take care of family responsibilities but have hurt their economic prospects, not to mention part-time jobs do not come with benefits such as health insurance.

Some racial-ethnic groups, particularly Black men, tend to suffer from higher unemployment rates and longer unemployment spells than non-Hispanic White workers [7]. Even when employed or having the same education as their White counterparts, Black workers tend to hold low-paying jobs with unpredictable and unstable hours, such as retail services or home health aides [19,26]. Black women are more likely than Black men or White women to experience not only difficulties in securing jobs but also low-paying jobs with unpredictable and unstable hours. Consequently, Black women overall experience a large pay gap, jeopardizing their economic security [20,26]. Even though the intersectionality of gender and race particularly disadvantages Black women in the workforce, this demographic has historically been more likely to be in paid work, albeit with low pay, than any other racial-ethnic group of women because the majority of them are breadwinners [20,26]. Extant research has shown that occupational segregation by race and gender, starting with one’s first employment, has put racial-ethnic group members on a cumulative disadvantaged pathway to holding jobs that are anything but steady work with decent wages and job-associated benefits [6,27], incurring lasting adverse consequences to their economic, physical, and psychological well-being [20,27].

Occupational segregation by race and gender is further compounded by the structural changes in the U.S. economy. For example, the broad shift from manufacturing jobs with stable full-time employment with job-associated benefits to unpredictable, unstable, and insecure employment since the 1980s has impacted every employment sector, but perhaps more so for those in service sectors (e.g., personal care, food preparation). An increasing number of workers experience persistently low wages while others reap considerable benefits by staying at the very top of the earnings distribution throughout their working lives [28], resulting in a polarized labor market. These labor market trends have disproportionately hurt nonwhite and women workers due to their high likelihood of holding low-paying jobs with increased insecurity.

#### 1.2.2. Work Schedules

In addition to these broad economic trends are the challenges of a shift toward nonstandard work schedules, hours outside of regular daytime hours (e.g., evenings, nights, or irregular hours). Most studies using both the U.S. and non-US samples on work schedules have primarily focused on the links between schedules and one’s physical, psychological, and social well-being [5,29] as well as on family and child well-being [10,30,31,32]. These studies have found adverse links between nonstandard work schedules and the well-being of workers and their families, particularly when such a schedule was chosen involuntarily (e.g., job requirements). Research has shown that workers in vulnerable positions, characterized by low education or low income, are more likely to be required to work nonstandard schedules [3,5]. Work patterns can have lasting impacts on the economic, physical, psychological, and social well-being of workers and their families, which translates into social stratification and inequality over generations. The results from these prior studies motivate the need to understand the prevalence and profile of people who work nonstandard schedules throughout their working years, those who transition into or out of such schedules, and those who rarely have nonstandard work schedules during their working lives.

A 2011 study by Presser and Ward [33] was the first to use data from the National Longitudinal Survey of Youth-1979 (NLSY79) to document employment trajectories from ages 18 to 39, paying particular attention to work schedules. That study found that working nonstandard hours (defined as a schedule other than a regular day shift) was common between ages 18 and 39, ranging between 20–30%. The prevalence was higher (around 50–60%) among those aged 18–22, perhaps due to working part-time while attending school. When accounting for the accumulated prevalence of working nonstandard hours, the results indicated that almost 90% of workers had at some point worked nonstandard hours by age 39, although again, this higher percentage might have attributed to the high prevalence among their early working years (aged 18–22). Men, Blacks, single parents, and those with a high school education or less were more likely than their corresponding counterparts to have ever worked nonstandard hours. Of note, women were more likely than men to have never worked or always worked nonstandard hours by age 39.

### 1.3. The Present Study

Presser and Ward’s 2011 study [33] offered a nuanced understanding from a longitudinal lens of the early years of one’s working life and how work patterns, particularly work schedules, may shape one’s career over time by gender, race-ethnicity, and education. Examining work schedules also provides insight into the rise in nonstandard work hours over our working lives and how this trend affects workers, including parents [10,34]. Because jobs with nonstandard work schedules tend to pay low wages and be unpredictable and unstable—all markers of a precarious job—studying work schedules is critical to understanding the prevalence of precarious employment [2,3]. Of note, our study examines the prevalence of having a nonstandard work schedule, a key indicator of a precarious job. We acknowledge that nonstandard work schedules are not exchangeable with an unpredictable and unstable job, although having a nonstandard work schedule increases the likelihood of having a jobs that is unpredictable and unstable job [14].

Using the longitudinal dataset of the NLSY79 from 1979 to 2018, our study adopts a person-driven statistical approach with refined work schedule patterns to provide a life course lens of the sampled youth’s working lives from ages 22 to 53 (the most recent available data point). Using prior literature, we identified several sociodemographic variables associated with groups likely to be engaged in nonstandard work schedules and those in vulnerable social positions [5]: gender, race-ethnicity, education, parenthood, and economic vulnerability (proxied by poverty and welfare status). Hence, an important contribution of our study is our specific attention to how, individually and jointly, each of the five social positions may be associated with a higher (lower) likelihood of engaging in work trajectories predominated by nonstandard schedules. We used sequence analysis to identify the most prevalent work schedule patterns over one’s working years before retirement. Parenthood was an important factor to include in the analyses because prior literature has consistently shown that nonstandard work schedules can be a challenge for parents juggling the demands of family and work [10,35]. Lastly, we used poverty and welfare status at or before age 23 as a proxy for whether individuals were in a vulnerable social position before starting their careers. Extensive literature has shown how these vulnerabilities put people on a disadvantaged trajectory throughout their lifetime [3,6,8,20].

## 2. Materials and Methods

### 2.1. Data

We used the National Longitudinal Survey of Youth-1979 (NLSY79) conducted by the U.S. Department of Labor. The NLSY79 contains a large, nationally representative sample of 12,686 men and women between the ages of 14 and 22 when first interviewed in 1979. The participants were interviewed annually until 1994 and then biennially thereafter. NLSY79 discontinued collecting data on two oversamples: the military youth discontinued in 1984 (1079 individuals were dropped but retained a sample of 201), and the non-Black non-Hispanic disadvantaged youths discontinued in 1990 (*n* = 1643). We excluded all 1280 oversampled military youths because they likely had different work schedule patterns than non-military participants. Excluding these two oversampled groups leaves a total of 9964 participants as our starting point. Despite the study following the same participants for about 40 years, the response rates have been remarkably high, from as high as 96% in the early years of the survey to about 77% in recent years [36]. The sample retained in the most recent year, 2018, was about 6800 [37]. The NYLS79 provides rich data, including histories of individuals’ sociodemographic characteristics (e.g., education, marriage, family structure, income) and annual work schedule. It remains the only study with a nationally representative sample that includes longitudinal information on work schedules over one’s working years.

We used age 22 as the starting point to document typical work schedule patterns over one’s working years for two reasons. First, as found in Presser and Ward’s study [33], about 40% of the surveyed people in the NLSY79 attended college between 18 and 22. During this period, they were especially likely to work nonstandard hours, possibly as part-time jobs. Age 22 is thus likely to be the starting point of establishing a career for many, particularly among those who graduated from college. Second, because participants ages 14 to 22 were interviewed in 1979 for the first time, the data set does not include information prior to 1979 for those aged 19–22 in 1979.

The survey year 2018 was the most recent publicly available data point when we conducted this study. We were thus able to document the work status for all participants from the age of 22 to 53. Specifically, we used 1987–2018 for those aged 14 in 1979. The corresponding years for those ages 15, 16, 17, 18, 19, 20, and 22 were, respectively, 1986–2016, 1985–2016, 1984–2014, 1983–2014, 1982–2012, 1981–2012, 1980–2010, and 1979–2010. As the NLSY79 conducted surveys biennially after 1994, for the purpose of sequence analysis, we replaced information in the non-interview years (e.g., 1995, 1997, 1999, etc.) with data from the prior year (e.g., 1994, 1996, 1998, etc.) to construct a work schedule by age. For instance, data on work schedules in 1995 were imputed from information in 1994 and so forth. We also conducted sensitivity analyses, setting the time axis as the survey year instead of age. The clustering pattern is nearly identical, suggesting our results are not sensitive to our approach of imputing information for non-interview years.

### 2.2. Participants

Our analysis includes respondents who have work schedule information from (1) at least one of the first two waves of survey years (ages 22 or 23) and (2) at least one of the last two waves of survey years (ages 50/51 or 52/53). Hereafter, we identified the working years from ages 22 to 53 for simplicity. About 16.32% of the 9964 respondents (*n* = 1593) in the NLSY79 were excluded due to not having any work schedule information at ages 50/51 or 52/53; less than 1% were excluded due to not having work schedule information at age 22/23 (*n* = 99); and another 2.86% were excluded due to missing work schedule information from both ages 22/23 and 50–53 (*n* = 285). These exclusions result in a total of 7987 respondents for our analysis (80.16% of 9964 respondents). This attrition rate is similar to those of other longitudinal surveys, such as the Panel Study of Income Dynamics [38]. Following previous literature, we did not conduct multiple imputations for missing cases due to attrition, a common practice adopted in studies using longitudinal panel data [39,40]. Finally, for another 9% of the 7987 respondents who had missing information on sociodemographic characteristics (the missing rate ranges from less than 0.02% on respondents’ education to 4.1% on whether the respondent has health conditions limiting work), we used multiple imputation to generate 10 imputated data sets to address missingness. Hence, our final analyzed sample size was 7987.

About half of the analyzed sample was males (vs. females) and about half identified as non-Hispanic White, one-third as non-Hispanic Black, 19% as Hispanic, and less than 1% as either Asian or other. About three-quarters of the people in the final analyzed sample were not considered to be immigrants, with an even split of 7–9% each identified as either first (i.e., the participant was not born in the U.S.), second (i.e., the participant was born in the U.S., but one or both parents of the participants were not born in the U.S.), or third-generation (i.e., both the participant and both parents were born in the U.S., but at least one of the grandparents were not born in the U.S.).

### 2.3. Measures

#### 2.3.1. Work Schedule Pattern

The NLSY79 surveyed participants at every assessment year regarding their work schedules. We followed the definitions and responses used by the NLSY to identify five work statuses for our sequence analysis. The NLSY coded work status as “standard” if work began at 6 a.m. or later and ended by 6 p.m., as “evenings” if the job began at 2 p.m. or later and ended by midnight, as “nights” if the job began at 9 p.m. or later and ended by 8 a.m., and as “variable” if the respondent had another type of schedule outside of standard hours (e.g., split shift, rotating shift, irregular hours). Those who were not working at any job at the time of the interview were identified as “not working.” We also included the category of “missing” for participants (about 3.88% of the 255,584 observations representing 7987 individuals across 32 years) who had missing information on work schedules during any of the assessment years after age 22 and before age 53.

#### 2.3.2. Social Position

We use five indicators to identify the participants’ social position. The first is gender, we used the information from 1979 as the primary data source to identify participants as either female or male (as the reference group). The second is race-ethnicity. We used responses from 1979 on questions asking separately about race and ethnicity to categorize respondents into the following five racial-ethnic groups: non-Hispanic White, non-Hispanic Black, Hispanic, Asian, and other. The third is education. The NLSY79 collected information on participants’ highest degree completed by the assessment year. We categorized respondent education levels into four groups based on the information at age 23: less than a high school degree (<12 years of schooling), a high school degree (12 years of schooling), some college (13–15 years of schooling), and college or higher (16+ years of schooling). We used a high school degree as the reference group for all analyses. The fourth is parenthood if the participant reported having any biological child by age 22/23. The fifth is poverty and welfare status. Previous studies have shown that people with low income are more likely to be engaged in precarious employment (e.g., low-wage, low-skill, nonstandard work schedules) [5]. Given that income and work are highly dependent on each other, we included in our analyses two dummy variables to capture aspects of poverty before or at age 23 (the beginning of our sample period). First, participants were considered to have ever lived in poverty by age 23 if they indicated that their family income was at 100% of or below the federal poverty threshold during the survey years before or at age 23. Second, the participants were considered to ever received welfare benefits by age 23 if they indicated they had ever received AFDC (Aids to Families with Dependent Children, the cash assistance prior to 1996 but was renamed to TANF after 1996), Food Stamps (in-kind assistance and was renamed to Supplemental Nutrition Assistance Program [SNAP] after 2008), or SSI (supplemental security income) at the age 23 or any previous survey years.

#### 2.3.3. Sociodemographic Characteristics

To address unobserved heterogeneity between participants, we considered a rich set of sociodemographic characteristics before or at age 23. These characteristics included age in 1979, immigrant status in 1979, parental education (i.e., less than high school, high school as the reference group, some college, or college and higher), residential location at age 14 (suburban, rural, versus urban), region of residence at age 22 (Northeast, Midwest, West, versus South), whether they were married at age 22/23, having a health condition limiting work by age 22/23, and occupation at age 22/23 (i.e., professional/managerial, sales-related, service-related, or other). Although the information on many of these characteristics was available at every assessment point, our analyses considered these characteristics before or at age 22/23 to address the possibility of reverse causality. As a robustness check, we conducted supplementary analyses in which we considered many of these sociodemographic characteristics after age 22/23 (e.g., marital status, parenthood, occupation); the results were similar to those presented here.

### 2.4. Data Analysis

We used Stata v.15 to perform all analyses. We first used sequence analysis to identify work schedule patterns between 22 and 53. A sequent analysis chronologically classifies the transitions of a categorical variable (or a state) and thus is well suited for analyzing changes in that state over time, in this case, work schedules [41]. To document the changes or transitions chronologically, the time axis in this analysis is each year between the ages of 22 and 53, and the state or categorical variable we tracked over time is the work schedule. Specifically, we used two steps to depict the trajectories of work schedules over the working years (i.e., sequences) and then clustered the trajectories into groups. First, we calculated the similarity and dissimilarity between sequences using an optimal matching algorithm by setting the “costs” of turning one sequence into another [42,43]. Following the literature, we set the insertion and deletion costs to be 1 and used the Needleman-Wunsch algorithm to calculate the substitution costs based on the transition rates between work schedule categories; when the transition is rare, the substitution cost is higher [44,45]. We conducted sensitivity analyses using alternative theoretical-driven substitution costs such as 2 or 3 [46]; the results indicate that our cluster patterns were not sensitive to the cost setting. Next, we clustered similar sequences into a finite number of groups using Ward’s hierarchical fusion algorithm [42]. We determined the ideal number of clusters using the stopping rules based on the Calinski and Harabasz pseudo-F index and the Duda-Hart index, as well as the conceptual meaning of clusters [47]. We present these diagnostic tests in Table S1. Of note, both sequence and latent class analyses fit well with the research questions examined here, and indeed both analyses yield similar results on classifications, producing equally reliable results with no preference for one over the other [48,49].

Next, we addressed the missingness with multiple imputation by creating 10 imputed data sets. We then performed a multinomial logit analysis for the multiple work schedule patterns identified through sequence analysis. In our multinomial logit regression, we used the cluster with the highest representation in the sample—working primarily standard hours—as the reference group.

Lastly, based on our multinomial logit regression results, we calculated predicted margins to see whether certain combinations of the five social positions (described above) might be more or less likely to exhibit specific work schedule patterns over the working years. We first computed marginal effects based on the multinomial logit results to predict probabilities of being in each work schedule pattern; we did so because multinomial logit models involve many comparisons and the reported coefficients can only provide the relative probability of a person having a specific work schedule pattern. We then calculated the predicted probabilities for various groups with multiple characteristics identified by gender, race-ethnicity, education by age 23, parenthood by age 23, poverty status by age 23, and welfare status by age 23. We present these predicted probabilities to show which group might have the highest likelihood of having a work schedule pattern characterized by nonstandard schedules, a key characteristic of a precarious job. All analyses considered all sociodemographic variables described in the Measures section.

## 3. Results

### 3.1. Descriptive Picture

**Figure 1:**
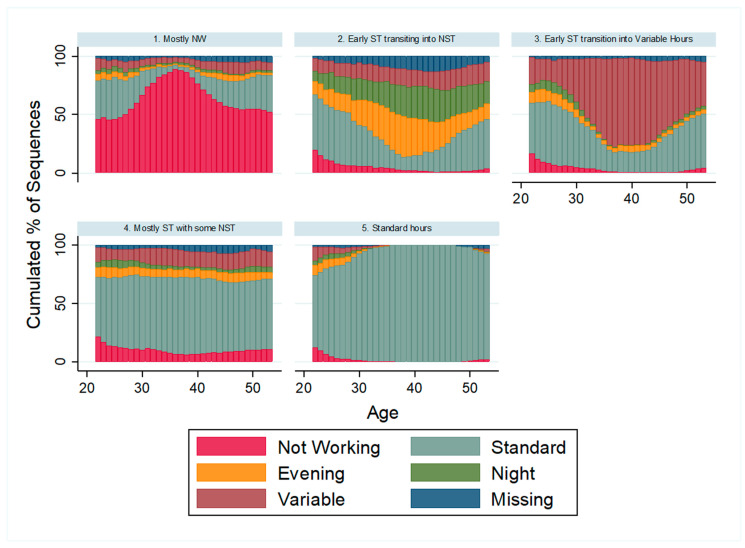
Distribution Plot of the Five Clusters.

Figure 1 presents the results from the sequence analysis. The five clusters represent the most parsimonious solution (based on the goodness-of-fit statistics presented in Supplementary Table S1). Specifically, given the distribution of the five work states within each cluster, we identified the first cluster as those mostly not working between ages 22 and 53, although they were most likely to have worked standard hours whenever they were employed. Cluster two comprises individuals who started working standard hours but soon, between ages 30 and 40, transitioned into nonstandard hours that were either evenings, nights, or variable. Cluster three comprises individuals with similar work schedule patterns as cluster two but began working predominately variable hours in their 30s. Cluster four contains individuals who mainly worked standard hours and a short period of nonstandard hours dominated by variable hours. Lastly, cluster five includes individuals primarily working standard hours throughout their working years between ages 22 and 53. Table 1 presents the percentage of the samples in each of these five clusters. About a quarter (25%) of the participants worked predominantly standard hours throughout their working years (cluster 5), and about 38% worked mainly standard hours with some degree of variable hours (cluster 4). Approximately 14% worked standard hours during the early years of their career but transitioned into variable hours (cluster 3), and about 13% worked standard hours early in their career but soon transitioned into the evening or nighttime hours (cluster 2). The remaining 10% were mostly not working between ages 22 and 52/53 (cluster 1). These findings demonstrate a remarkably high prevalence (65%) of working nonstandard hours to varying degrees (clusters 2–4) between ages 22 and 53 in the United States.

Table 1 presents descriptive information on all of our analyzed variables for the total sample as well as separately by the five clusters identified through the sequence analysis. In general, this nationally representative cohort of young men and women ages 14–22 in 1979 came from diverse backgrounds in terms of gender, race-ethnicity, parental education, and their own educational achievement. Across the five clusters, raw data suggest that people who were mostly not working between ages 22 and 53 were more likely to be female, non-Hispanic Black or Hispanic, have less than high school education, be a parent by age 23, have health conditions limiting their work, have lived in poverty at some point by age 23, have ever received welfare by age 23, and have parents whose highest education was less than high school. In contrast, individuals whose work schedule patterns identified as mostly standard hours with some nonstandard schedules (cluster 4) or predominately standard hours (cluster 5) tended to have the opposite characteristics as those mostly not working (cluster 1). Of importance, non-Hispanic Blacks and those with low levels of education (both their own and their parents) (e.g., high school degree or below) tended to fall in cluster 2, working standard hours during the early years but transitioning quickly into nonstandard hours; this group also had the highest prevalence of working either evening or night hours versus working variable hours.

### 3.2. Characteristics Contributing to Different Work Schedule Patterns

Table 2 presents the multinomial logit regression results where the five clusters were the outcome categories, with cluster 5 as the reference group. Results in Table 2, in general, are consistent with the descriptive picture presented in Table 1. Specifically, females, non-Hispanic Blacks, those with low educational attainment (high school or below) at age 23, the types of occupation at ages 22/23, those with health conditions that limited their ability to work by age 23, and poverty and welfare experiences by age 23 were consistently significantly associated with individuals’ work patterns between ages 22 and 53. Because multinomial logit models involve many comparisons and the reported coefficients in these models can only provide the relative probability of being in various work schedule patterns, we next present Table 3 with predicted probabilities for groups of interest based on the multinomial logit results reported in Table 2. We first show the predicted probabilities for each variable of interest identified by gender, race-ethnicity, education, parenthood, poverty, and welfare status. We then present the predicted probabilities by combining these characteristics to show which sociodemographic groups have the highest or the lowest likelihood of having specific work schedule patterns throughout their working years. For brevity, we present the comparisons between non-Hispanic White and non-Hispanic Black. The probabilities for Hispanics of having certain work schedule patterns tend to lie between those for non-Hispanic White and non-Hispanic Black. Due to extremely small sample sizes, we do not present probabilities for Asians or those in the other racial category.

The first row of Table 3 presents the predicted probabilities for the reference group based on the multinomial logit model reported in Table 2. Consistent with the descriptive information, our analyzed sample of young men and women ages 14–22 in 1979 had a 0.29 likelihood of working standard hours (cluster 5) throughout their working years up until age 53. They had a 0.34 likelihood of working mostly standard hours with some nonstandard work hours (cluster 4). This sample had an approximate likelihood of 0.15–0.19 of having work schedule patterns identified as clusters 2 and 3, working standard hours early in their working life before switching to either working evening/night hours or mainly working variable hours. Finally, they had a likelihood of 0.03 of falling within cluster 1, mostly not working between ages 22 and 53.

Table 3 also presents the kind of work schedule pattern likely experienced by different sociodemographic groups. For example, being a female and having less than a high school education was associated with approximately a 0.07 and 0.06 probability, respectively, of mostly not working throughout one’s working years, double the likelihood of the reference group. In comparison, those with a college degree or above had a 0.43 probability of working standard hours throughout their working years (cluster 5). In the following subsections, we discuss the predicted probabilities for each of the five clusters, with intersections of various sociodemographic characteristics related to gender, race-ethnicity, education, parenthood, and poverty and welfare status.

*Mostly not working (cluster 1)*: As shown in Table 3, non-Hispanic Black mothers with less than a high school education who had lived in poverty and received welfare at some point by age 23 had the highest likelihood (0.21) of not working among all participants, followed by non-Hispanic White mothers with the same characteristics of education, poverty, and welfare status (a likelihood of 0.16). In contrast, non-Hispanic White and Black males with a college degree or above who were not parents and who had never lived in poverty nor received welfare by age 23 had the lowest likelihood of mostly not working (0.02 and 0.03, respectively).

*Working standard hours early on but transitioning to nonstandard hours (cluster 2)*: The predicted probabilities shown in Table 3 indicate that non-Hispanic Black males had the consistently highest likelihood among all subgroups to work standard hours during early years but soon transition to working evening or night hours. Neither having low education (high school degree or below), being a parent, nor having experienced poverty or received welfare changes the probabilities. These results suggest that the social position of Black males was a strong force behind having such a work schedule pattern. Two groups had the highest likelihood of falling within cluster 2: non-Hispanic Black fathers with high school education and non-Hispanic Black males with a high school education who had ever received welfare by age 23 (likelihoods of 0.23). In contrast, the lowest probability of having this work schedule pattern is among non-Hispanic White males and females and non-Hispanic Black females with a college degree, who were not parents at age 23, and had never lived in poverty nor received welfare by age 23 (a probability of 0.05 to 0.08). Of importance, the probability was 0.11 for non-Hispanic Black males even when they had similarly advantaged backgrounds as non-Hispanic White males and females.

*Working standard hours early but transitioning to mainly variable hours (cluster 3)*: As shown in Table 3, two groups had the highest probabilities of having this work schedule pattern: people with some college education (a probability of 0.24) and people who had received welfare at some point by age 23 (a probability of 0.22). In contrast, non-Hispanic White and Black mothers with less than a high school education and who had ever experienced poverty and received welfare by age 23 had the lowest likelihood of engaging in this work schedule pattern (a probability of 0.08 to 0.11). These somewhat contradictory results could be related to how the NLSY79 defines “variable hours.” While such hours are nonstandard, this work schedule category may comprise a heterogeneous group that includes a diverse set of occupations and both those who voluntarily chose such a work schedule and those forced to work variable hours.

*Mainly working standard hours with some nonstandard hours (cluster 4)*: Table 3 indicates that the broad groups most likely to have such a work schedule pattern were women (a probability of 0.41) and those with less than a high school education (0.39). Of importance, non-Hispanic Blacks with less than a high school education had a high likelihood of having such a work schedule pattern, particularly when they were fathers and had, at some point by age 23, experienced poverty (ranging from 0.44 to 0.45). The groups with the highest likelihood of falling within cluster 4 were non-Hispanic Black mothers and non-Hispanic White mothers with less than a high school education and ever experienced poverty and welfare (a probability of 0.47–0.48). Non-Hispanic White and Black fathers had the next highest probability (0.45) of falling within cluster 4.

*Working standard hours throughout working years (cluster 5)*: Results in Table 3 indicate that having a college or above education was a significant factor in having this work schedule pattern, with a probability of 0.43 that does not vary by other characteristics such as gender or parenthood. Thus, non-Hispanic White males with a college degree or above who were never poor and had never received welfare by age 23 had the highest likelihood of having such a work schedule (0.43), followed by non-Hispanic White females with the same characteristics (0.40). Non-Hispanic Black males and females with these same characteristics also had a high likelihood of having such a work schedule pattern (0.35 and 0.32, respectively), albeit at lower levels than non-Hispanic White males and females. In contrast, the groups with the lowest likelihood (0.11) of having this work schedule pattern were non-Hispanic Black mothers with less than a high school education who had lived in poverty or received welfare at some point by age 23. Non-Hispanic Black fathers and non-Hispanic White mothers with these same characteristics had a similarly low probability (0.13–0.15) of having such a work schedule pattern.

## 4. Discussion

We set out to document the working lives of young men and women aged 14–22 in 1979 to understand the prevalence of having a nonstandard work schedule throughout their working years in the United States. A nonstandard work schedule, a key indicator of precarious employment, has become a common job arrangement in our polarized and globalized labor market [3,4]. As the world has battled an ongoing public health crisis, we have witnessed much of the harm being disproportionately shouldered by people without resources who tend to hold low-wage, low-skill, or precarious jobs [50], particularly in the United States [51]. Our study sheds important light on the groups most likely to work nonstandard hours, and thus most likely to have a vulnerable work trajectory.

Examining work status from age 22 to 53, we found five general clusters describing the majority of our workforce. As expected, work schedule patterns from ages 22 to 53 were stratified by gender, race-ethnicity, education, and vulnerable economic status. Specifically, being female, being non-Hispanic Black, having low educational attainment (e.g., high school or below), and having lived in poverty or received welfare at some point by age 23 all increased the likelihood of engaging in work schedule patterns other than standard hours throughout their working years. Below we highlight several results.

First, we found that low-educated non-Hispanic Black males who had experienced poverty or received welfare at some point before age 23 had the highest likelihood of falling within cluster 2, characterized by working standard hours during early years but transitioning to working at either evening or night hours between ages 30 and 50. Although not shown, our raw data indicate that the annual wages earned by people in each of the five clusters appear to be in a ranking hierarchy, with people holding cluster 5 work schedule patterns earning the highest wages. Those in cluster 2 (and also cluster 1) earned substantially less than those in clusters 5, 4, and 3. Within each cluster, people holding professional/managerial occupations earned the highest wages, followed by those with sales jobs, and then by occupations in either services or other categories. Of importance, people working in the service sectors had the lowest annual wages, with a work schedule that started with standard hours but soon transitioned to working mainly evening or night hours. The sociodemographic characteristics associated with a high likelihood of having a cluster 2 work schedule pattern clearly show that these people were in a vulnerable position before entering the workforce, which made them likely to end up in a work pattern characterized by precarious jobs (e.g., low wage, low skills, with a few or no benefits). Our findings thus suggest that our labor market arrangements have increasingly perpetuated the cumulative effects of vulnerabilities or inequalities stemming from social position, which, in turn, shape one’s life opportunities [52].

Second, our findings regarding the work schedule pattern identified as cluster 3 suggest that respondents in the NLSY79 sample may work variable hours for different reasons. For example, having some college education was significantly associated with starting a career working standard hours and then transitioning into mainly variable hours between the ages of 30 and 50. Yet, we also found that a group with a high likelihood of engaging in such a work schedule was non-Hispanic Black males with a high school degree who had received welfare before age 23 (0.19). Our data indicate that people in this cluster tended to hold either professional/managerial or service occupations. The former likely chose to work variable hours voluntarily, whereas the latter likely had no choice. A representative group of the former is the highly educated non-Hispanic White males who neither experienced poverty nor received welfare before age 23, having a 0.18 probability of engaging in a work schedule pattern identified as cluster 3. This group tended to work in professional or managerial occupations, suggesting that variable hours might have been a voluntary work arrangement. For the latter group, previous research has indicated that when chosen involuntarily, variable hours tend to be a catch-all term to capture someone working either split, rotating, or irregular shifts; such a schedule tends to bring uncertainty to our daily routine, including arranging family responsibilities [5,10]. However, we acknowledge that it is beyond the scope of the paper to speak with certainties.

Third, we found that non-Hispanic Blacks with less than a high school education tended to have a high likelihood of engaging in a work schedule pattern characterized by working mostly standard hours with some nonstandard hours (cluster 4). This likelihood was even more pronounced for parents or those who had experienced poverty or received welfare at some point by age 23. Their occupations tended to be evenly split between professional/managerial, sales, services, and others. Although beyond the scope of this analysis, this group of workers might be characterized as beating the odds of their vulnerabilities (i.e., being a racial-ethnic minority who had experienced poverty or received welfare before adulthood) to secure a work schedule pattern with mostly standard hours. This group worked some variable hours during their working years, though it is unclear from the data whether this was required or chosen voluntarily. However, one of the tenets of cumulative advantage/disadvantage (CAD) theory is that although opportunities beget opportunities, constraints or vulnerabilities do not necessarily hinder opportunities [52]. A detailed examination of the wage and income profiles over time of people who fall within cluster 4 may shed light on how such a work schedule pattern shaped their life course in a positive trajectory. Understanding what factors contributed to a person falling within cluster 4 versus clusters 1 and 2 despite sharing similar early life disadvantages (e.g., poverty and welfare experience) could point to modifiable factors to avoid precarious work schedule trajectories.

### Limitation

Our study is bound to have limitations. One of the significant limitations is, due to the data at hand, our inability to clearly distinguish the meaning of “variable hours” in the NLSY79. This was further constrained by NLSY categorizing shifts of split, rotating, and irregular into a single category of “variable hours,” preventing us from conducting analyses with more refined categories of nonstandard work schedules. As attested by previous studies [5], whether a person works variable hours voluntarily or involuntarily has significant implications for understanding the vulnerability associated with the work schedule patterns we discovered here. Nonetheless, the sociodemographic profiles associated with work schedule patterns involving variable hours provide much-needed insight into how variable hours may underscore opportunities versus constraints for different sociodemographic groups. Another limitation is that our analyses did not examine partners’ joint work schedule patterns. Extant research has shown that parents arrange their work schedules jointly with their spouses to meet family needs and childcare demands [53,54]. Third, although our analysis has deliberately kept the racial-ethnic group of Asians and others as a separate group for their low or none representation even in a national dataset, our analysis unfortunately still suffers the extremely small sample sizes, prohibiting us from making any definite statements about these racial groups. Fourth, NLSY79 only provided work schedule information biennially after 1994. Work schedules may have changed during those two years, limiting the precision of our work schedule trajectories over time. However, our longitudinal approach to identifying work trajectories in actuality may reduce measurement noises. Longitudinal data allow more accuracy in identifying, for example, someone who has reported repeatedly working nonstandard hours versus those who might have only worked a few times over the working years. Fifth, analyses combining both schedules and hours (e.g., full-time vs. part-time) would provide a richer picture of employment trajectories than what is presented here. However, in our sample, the number of observations of people working standard hours and working part-time was extremely small (e.g., 2% cases), thus not allowing for meaningful cluster classifications. This finding suggests that even with a large sample such as the NLSY79, analyses are still compromised by small cell sizes due to refined categories. Notwithstanding this limitation, NLSY79 is the only nationally representative dataset that has collected work schedule information across more than three decades. Lastly, despite the sequence analysis allowing for a more dynamic approach to capturing transitions over the participants’ working lives, it is beyond the scope of this paper to examine how social position or earlier work schedule patterns may have shaped the dynamic changes or transitions throughout working years or at later time points, a topic that warrants future analysis.

## 5. Conclusions

Mindful of these limitations, our study sheds much-needed light on how work-schedule patterns might shape individuals’ employment history in the United States. Given that precarious jobs have become increasingly common in this globalized and polarized labor market and that nonstandard work schedules are a defining feature of precarious jobs, examining work schedule patterns through a longitudinal lens also allows for a deeper appreciation of how the prevalence of certain schedules might be a manifestation of the cumulated advantages and disadvantages over one’s working life. Opportunities beget opportunities. The groups that hold vulnerable social positions in our study also tended to have jobs that required nonstandard work schedules, further aggravating their chances of accessing opportunities and resources to alter the course of their employment trajectories. Our analysis thus presents a sobering look at how our work as a social system may generate and perpetuate vulnerabilities and thus inequalities that manifest not only over the life course but possibly across generations.

Taken together, our sequence analysis speaks volumes to how early life vulnerabilities may matter to our lifelong work and well-being. Specifically, experiencing poverty and welfare assistance before adulthood, markers for early life vulnerabilities, significantly increases one’s probability of engaging in lifelong patterns of nonstandard work schedules, a key indicator for a precarious job that brings low wages, unpredictable hours and earnings, and little to no benefits. Thus, our results hold significant implications for developing and expanding policies to alleviate the adverse consequences of early life vulnerabilities on entering into precarious work trajectories in adult life. For example, to address childhood hardship including poverty, studies have found that income-support policies tend to incur the highest returns to better education and health outcomes during childhood [55,56], consequentially reducing vulnerabilities for unfavorable lifelong work patterns such as those documented here. In addition, to properly support workers’ challenges in meeting family and work demands, governments can incentivize or subsidize employers to institute flexible work arrangements and family leave policies to reduce the struggles faced by workers with unpredictable and unstable work hours. Finally, employment policies that guarantee living wages (instead of a minimum wage) would ensure decent wages for sustainable living standards to support workers, their families, and children, thus reducing cross-generational inequality [57].

## Figures and Tables

**Table 1:** Descriptive Statistics for Overall Sample and by Cluster.

	Total	1. Mostly NW	2. Early ST Transiting into NST	3. Early ST Transiting into Variable Hours	4. Mostly ST with Some NST	5. Standard Hours	χ^2^ or F
N	7987 (100%)	833 (10.43%)	1029 (12.88%)	1109 (13.89%)	3055 (38.25%)	1961 (24.55%)	
Characteristics	Mean or %	Mean or %	Mean or %	Mean or %	Mean or %	Mean or %	
Age	17.62	17.55	17.33	17.47	17.33	17.58	4.05 **
Female	51.16%	14.49%	10.96%	12.09%	40.33%	22.12%	205.07 ***
Male	48.84%	6.18%	14.89%	15.77%	36.07%	27.10%	
Race-ethnicity							236.48 ***
Non-Hispanic White	49.82%	7.67%	10.91%	16.34%	36.39%	28.70%	
Non-Hispanic Black	30.36%	13.94%	16.82%	11.67%	39.88%	17.69%	
Hispanic	19.24%	11.97%	11.97%	11.06%	40.60%	24.40%	
Asian	0.59%	4.26%	8.51%	6.38%	29.79%	51.06%	
Other races	0.51%	12.20%	9.76%	14.63%	41.46%	21.95%	
Immigration status							42.93 **
Not an immigrant	75.44%	10.44%	13.18%	14.22%	38.36%	23.80%	
First generation	7.62%	12.32%	13.30%	8.87%	39.90%	25.62%	
Second generation	9.09%	12.40%	11.71%	15.56%	34.71%	25.62%	
Third generation	7.85%	6.22%	11.00%	13.56%	39.71%	29.51%	
Education by age 23							489.35 ***
Less than high school	20.46%	20.66%	12.69%	8.89%	40.96%	16.80%	
High school degree	43.26%	9.22%	15.69%	13.28%	38.85%	22.96%	
Some college	22.87%	6.91%	11.57%	17.99%	37.52%	26.00%	
College degree or above	13.41%	4.68%	6.08%	16.46%	33.77%	39.01%	
Married at age 22/23	36.49%	10.36%	12.80%	13.73%	39.09%	24.02%	1.49
Not married at age 22/23	63.51%	10.47%	12.91%	13.98%	37.78%	24.86%	
Parenthood by age 23	33.89%	14.59%	13.82%	11.49%	41.19%	18.91%	150.00 ***
Not a parent by age 23	66.11%	8.30%	12.41%	15.11%	36.74%	27.44%	
Occupation at age 22/23							762.86 ***
Not working	14.56%	29.49%	12.64%	9.54%	38.61%	9.72%	
Professional	18.99%	4.68%	8.50%	17.53%	35.07%	34.21%	
Sales	5.33%	9.39%	8.45%	17.14%	39.91%	25.12%	
Services	35.97%	9.19%	14.31%	14.24%	39.54%	22.73%	
Other Occupation	25.14%	5.73%	15.24%	12.45%	38.25%	28.34%	
Have health conditions limiting work before age 22/23	9.62%	16.82%	13.03%	14.11%	40.30%	15.74%	66.889 ***
Not having health conditions limiting work before age 22/23	90.38%	9.61%	12.90%	13.87%	38.12%	25.51%	
Ever in poverty by age 22/23	44.57%	14.31%	13.39%	11.55%	40.92%	19.83%	188.42 ***
Never in poverty by age 22/23	55.43%	7.28%	12.62%	15.87%	35.93%	28.30%	
Ever received welfare by age 22/23	22.06%	20.05%	14.08%	11.24%	40.49%	14.14%	316.89 ***
Never received welfare by age 22/23	77.94%	7.70%	12.55%	14.64%	37.60%	27.51%	
Parents’ highest education							180.62 ***
Less than high school	34.06%	13.47%	14.69%	10.34%	40.94%	20.56%	
High school degree	39.19%	8.85%	12.47%	14.99%	37.93%	25.76%	
Some college	11.55%	8.55%	12.71%	15.07%	35.21%	28.46%	
College degree or above	15.20%	6.67%	8.89%	19.57%	35.64%	29.23%	
Residence at age 14							17.73 *
Urban	79.15%	10.82%	12.89%	13.71%	38.44%	24.14%	
Suburban	15.75%	9.46%	12.56%	13.20%	38.63%	26.15%	
Rural	4.75%	7.39%	12.93%	19.00%	34.04%	26.65%	
Region at age 22/23							30.95 **
South	40.26%	9.61%	13.13%	13.07%	40.55%	23.64%	
Northeast	19.31%	12.55%	13.26%	12.81%	36.28%	25.01%	
Midwest	24.51%	9.94%	14.09%	15.11%	35.55%	25.31%	
West	21.02%	10.39%	11.23%	14.87%	39.13%	24.37%	

Note. *** *p* < 0.001, ** *p* < 0.01, * *p* < 0.05. The Chi-square and anova tests were based on randomly selected one imputation because the -mi- command in Stata does not support these tests.

**Table 2:** Multinomial Regression Estimates of Work Patterns (N = 7987).

Reference: 5. Standard Hours	1. Mostly NW	2. Early ST Transiting into NST	3. Early ST Transiting into Variable Hours	4. Mostly ST with Some NST
Age	−0.024	−0.055 **	−0.015	−0.045 **
	(0.021)	(0.019)	(0.018)	(0.014)
Female (Ref: Male)	0.983 ***	−0.195 *	−0.229 **	0.252 ***
	(0.109)	(0.091)	(0.086)	(0.068)
Race-ethnicity (Ref: Non-Hispanic White)				
Non-Hispanic Black	0.604 ***	0.660 ***	0.107	0.334 ***
	(0.119)	(0.104)	(0.105)	(0.082)
Hispanic	0.060	0.043	−0.206	0.086
	(0.148)	(0.134)	(0.130)	(0.098)
Asian	−1.329 +	−0.812	−1.538 *	−0.798 *
	(0.767)	(0.558)	(0.625)	(0.352)
Other Race-ethnicity	−0.204	−0.136	0.099	0.137
	(0.62)	(0.616)	(0.538)	(0.429)
Immigration Status (Ref: Not an immigrant)				
First generation	−0.039	0.065	−0.322+	−0.074
	(0.185)	(0.168)	(0.183)	(0.126)
Second generation	0.109	−0.122	0.135	−0.208 +
	(0.158)	(0.150)	(0.139)	(0.113)
Third generation	−0.256	−0.052	−0.194	0.051
	(0.197)	(0.155)	(0.143)	(0.108)
Education by age 23 (Ref: High school)				
Less than high school	0.912 ***	−0.004	−0.015	0.284 **
	(0.119)	(0.115)	(0.125)	(0.091)
Some college	−0.525 ***	−0.383 ***	0.114	−0.154 +
	(0.132)	(0.107)	(0.100)	(0.081)
College degree or above	−0.928 ***	−1.173 ***	−0.450 **	−0.520 ***
	(0.195)	(0.166)	(0.131)	(0.104)
Married at age 22/23	0.016	−0.025	0.045	−0.033
	(0.108)	(0.098)	(0.094)	(0.074)
Parenthood by age 23	−0.393**	0.070	−0.053	0.039
	(0.127)	(0.112)	(0.113)	(0.086)
Occupation at age 22/23 (Ref: Services)				
Not working	1.797 ***	0.548 ***	0.394 **	0.734 ***
	(0.140)	(0.145)	(0.152)	(0.120)
Professional	−0.492 **	−0.492 ***	−0.100	−0.238 **
	(0.158)	(0.126)	(0.107)	(0.086)
Sales	0.214	−0.405	0.123	0.077
	(0.207)	(0.206)	(0.167)	(0.136)
Other Occupation	−0.425 **	−0.321 **	−0.422 ***	−0.204 *
	(0.141)	(0.108)	(0.110)	(0.084)
Have health conditions limiting work by age 22/23	0.718 ***	0.294 *	0.410 **	0.334 **
	(0.150)	(0.149)	(0.145)	(0.118)
Ever in poverty by age 22/23	0.293 **	0.024	−0.034	0.172 *
	(0.107)	(0.092)	(0.090)	(0.069)
Ever received welfare by age 22/23	0.538 ***	0.309 *	0.383 **	0.237 *
	(0.129)	(0.119)	(0.122)	(0.095)
Parents’ highest education (Ref: High school)				
Less than high school	0.093	0.195+	−0.149	0.069
	(0.115)	(0.100)	(0.105)	(0.078)
Some college	0.180	0.166	−0.046	−0.025
	(0.162)	(0.135)	(0.126)	(0.101)
College degree or above	0.353 *	0.176	0.310 **	0.173+
	(0.165)	(0.140)	(0.115)	(0.097)
Residence at age 14 (Ref: Urban)				
Suburban	−0.158	−0.114	−0.099	−0.095
	(0.128)	(0.111)	(0.108)	(0.083)
Rural	−0.304	−0.029	0.309+	−0.159
	(0.236)	(0.185)	(0.164)	(0.142)
Region at age 22/23 (Ref: South)				
Northeast	0.380 **	0.136	−0.065	−0.046
	(0.130)	(0.117)	(0.115)	(0.088)
Midwest	0.227+	0.290 **	0.104	0.008
	(0.126)	(0.108)	(0.104)	(0.083)
West	0.303 *	0.049	0.156	0.055
	(0.133)	(0.119)	(0.111)	(0.087)
Constant	−1.835 ***	0.307	−0.157	0.966 ***
	(0.415)	(0.358)	(0.344)	(0.269)

Note. *** *p* < 0.001, ** *p* < 0.01, * *p* < 0.05, + *p* < 0.10.

**Table 3:** Predicted Probability of Work Schedule Patterns by Various Characteristics.

	1. Mostly NW	2. Early ST Transiting into NST	3. Early ST Transiting into Variable Hours	4. Mostly ST with Some NST	5. Standard Hours
Reference group	0.03	0.15	0.19	0.34	0.29
1. Male	0.03	0.15	0.19	0.34	0.29
2. Female	0.07	0.11	0.14	0.41	0.26
3. Non-Hispanic White	0.03	0.15	0.19	0.34	0.29
4. Non-Hispanic Black	0.04	0.22	0.16	0.36	0.22
5. Hispanic	0.03	0.15	0.16	0.37	0.29
6. Less than high school (LTHS)	0.06	0.13	0.16	0.39	0.25
7. High school degree (HS)	0.03	0.15	0.19	0.34	0.29
8. Some college (SC)	0.02	0.11	0.24	0.32	0.31
9. College degree or above (College+)	0.02	0.07	0.18	0.30	0.43
10. Being a parent by age 23 (parent)	0.02	0.16	0.18	0.35	0.29
11. Ever in poverty by age 23 (poor)	0.04	0.14	0.17	0.38	0.27
12. Ever received welfare by age 23 (welfare)	0.04	0.16	0.22	0.34	0.23
Potential Vulnerable Groups					
1 + 4: Black male	0.04	0.22	0.16	0.36	0.22
1 + 4 + 6: Black male with LTHS	0.09	0.19	0.13	0.41	0.18
1 + 4 + 7: Black male with HS	0.04	0.22	0.16	0.36	0.22
1 + 4 + 8: Black male with SC	0.03	0.17	0.21	0.35	0.25
1 + 4 + 9: Black male with College+	0.03	0.11	0.17	0.35	0.35
1 + 4 + 6 + 10: Black fathers parent with LTHS	0.06	0.20	0.13	0.43	0.19
1 + 4 + 7 + 10: Black fathers with HS	0.03	0.23	0.15	0.37	0.22
1 + 4 + 6 + 11: Black male with LTHS and poor	0.11	0.17	0.12	0.44	0.17
1 + 4 + 6 + 12: Black male with LTHS and welfare	0.11	0.19	0.15	0.40	0.14
1 + 4 + 7 + 11: Black male with HS and poor	0.05	0.20	0.14	0.40	0.20
1 + 4 + 7 + 12: Black male with HS and welfare	0.05	0.23	0.19	0.36	0.17
1 + 4 + 6 + 10 + 11: Black fathers with LTHS and poor	0.07	0.18	0.11	0.46	0.17
2 + 4 + 6 + 10 + 11 + 12: Black mothers with LTHS and poor, receiving welfare	0.21	0.13	0.08	0.47	0.11
1 + 4 + 6 + 10 + 11 + 12: Black fathers with LTHS and poor, receiving welfare	0.10	0.19	0.13	0.45	0.13
2 + 3 + 6 + 10 + 11 + 12: White mothers with LTHS and poor, receiving welfare	0.16	0.10	0.11	0.48	0.15
1 + 3 + 6 + 10 + 11 + 12: White fathers with LTHS and poor, receiving welfare	0.07	0.14	0.16	0.45	0.18
Potential Non-Vulnerable Groups					
2 + 4 + 9: Black female with College+ and never poor nor receiving welfare	0.06	0.08	0.12	0.41	0.32
1 + 4 + 9: Black male with College+ and never poor nor receiving welfare	0.03	0.11	0.17	0.35	0.35
2 + 3 + 9: White female with College+ and never poor nor receiving welfare	0.04	0.05	0.14	0.37	0.40
1 + 3 + 9: White male with College+ and never poor nor receiving welfare	0.02	0.07	0.18	0.30	0.43

Note: Margins were calculated based on multinomial logit results presented in Table 2. The reference group is male, non-Hispanic White, not an immigrant, living in an urban area at age 14, and with the following characteristics at ages 22/23: received a high school degree, not married, not a parent, working in the service sector, not having health limitation, never in poverty, never receiving welfare, parents’ highest education being high school, and living in the South.

## Data Availability

The data set, on which the findings of the study are based, is publicly available at https://www.bls.gov/nls/nlsy79/using-and-understanding-data/home.htm (accessed on 31 March 2021.

## Supplementary Materials

The following supporting information can be downloaded at: https://www.mdpi.com/article/10.3390/ijerph192013677/s1, Table S1: Goodness-of-fit Statistics for Cluster Solutions. Reference [58] is cited in the supplementary materials.
