# Supplementary material for: Changing Employment and Work Schedule Patterns over the 30 Working Years—A Sequential Cluster Analysis
Source: Int J Environ Res Public Health. 2022 Oct 21;19(20):13677. doi: 10.3390/ijerph192013677 (PMC9603687; doi:10.3390/ijerph192013677)
Supplement: Supplementary file 1 [file ijerph-19-13677-s001.zip › ijerph-1972006-supplementary.pdf]

Supplementary Material

Table S1. Goodness-of-fit Statistics for Cluster Solutions

Number of Clusters	Calinski/Harabasz pseudo-F	Duda/Hart	
		Je(2)/Je(1)	Pseudo T-squared
2	10.43	0.9953	13.95
3	12.22	0.9982	9.27
4	11.24	0.9874	27.22
5	15.31	0.9998	0.71
6	12.39	0.9998	0.50
7	10.41	0.9989	0.91
8	9.05	0.9995	0.55
9	7.98	0.9966	3.52
10	7.51	0.9994	1.02
11	6.86	1	0.01
12	6.24	0.9989	1.01
13	5.80	0.9991	0.74
14	5.41	0.9938	6.30
15	5.48	0.9977	1.87

Note. The two stopping-rule values for each cluster solution are presented above, which are the Calinski and Harabasz pseudo-F index and the Duda-Hart Je(2)/Je(1) index. In both indices, larger values indicate a more distinct cluster. However, smaller pseudo-T-squared values indicate more distinct clustering (Milligan and Cooper, 1985).

Milligan, GW.; Cooper, MC. An examination of procedures for determining the number of clusters in a data set. *Psychometrika*. **1985**; *50*(2): 159-179. https://doi.org/10.1007/BF02294245
